# Time trends and risk factor associated with premature birth and infants deaths due to prematurity in Hubei Province, China from 2001 to 2012

**DOI:** 10.1186/s12884-015-0767-x

**Published:** 2015-12-10

**Authors:** Haiqing Xu, Qiong Dai, Yusong Xu, Zhengtao Gong, Guohong Dai, Ming Ding, Christopher Duggan, Zubin Hu, Frank B. Hu

**Affiliations:** Department of Child Health Care, Hubei Maternal and Child Health Hospital, Wuhan, China; Department of Nutrition, Harvard School of Public Health, 655 Huntington Ave, Boston, MA 02115 USA; Boston Children’s Hospita, Boston, Massachusetts USA

## Abstract

**Background:**

The nutrition and epidemiologic transition has been associated with an increasing incidence of preterm birth in developing countries, but data from large observational studies in China have been limited. Our study was to describe the trends and factors associated with the incidence of preterm birth and infant mortality due to prematurity in Hubei Province, China.

**Methods:**

We conducted a population-based survey through the Maternal and Child Health Care Network in Hubei Province from January 2001 to December 2012. We used data from 16 monitoring sites to examine the trend and risk factors for premature birth as well as infant mortality associated with prematurity.

**Results:**

A total of 818,481 live births were documented, including 76,923 preterm infants (94 preterm infants per 1,000 live births) and 2,248 deaths due to prematurity (2.75 preterm deaths per 1,000 live births). From 2001 to 2012, the incidence of preterm birth increased from 56.7 to 105.2 per 1,000 live births (*P* for trend < 0.05), while the infant mortality rate due to prematurity declined from 95.0 to 13.4 per 1,000 live births (*P* for trend < 0.05). Older maternal age, lower maternal education, use of assisted reproductive technology (ART), higher income, residence in urban areas, and infant male sex were independently associated with a higher incidence of preterm birth (all *p* values < 0.05). Shorter gestation, lower birth weight, and lower income were associated with a higher mortality rate, while use of newborn emergency transport services (NETS) was associated with a lower preterm mortality rate (all *p* values < 0.05).

**Conclusion:**

An increasing incidence of preterm birth and a parallel reduction in infant mortality due to prematurity were observed in Hubei Province from 2001 to 2012. Our results provide important information for areas of improvements in reducing incidence and mortality of premature birth.

**Electronic supplementary material:**

The online version of this article (doi:10.1186/s12884-015-0767-x) contains supplementary material, which is available to authorized users.

## Background

Approximately 15 million infants are born prematurely each year, which is more than one tenth of all new-born infants globally [1]. Preterm infants have a high risk of birth complications, including infectious diseases, respiratory insufficiency, intraventricular hemorrhage, neurosensory deficits, and other organ system involvement [2]. Mortality due to complications of prematurity is the leading cause of neonatal death, and the second leading cause of death for children under age five [3]. Achieving Millenium Development Goal to reduce child mortality is therefore in large part dependent on reducing mortality related to premature birth [4].

For preterm infants who do survive, elevated risks for cognitive disorders and chronic non-communicable diseases exist [5, 6]. Preterm birth has been associated with elevated plasma insulin levels [7], altered growth patterns [5], and higher risk for cardiovascular diseases in adulthood [5]. The incidence of prematurity and subsequent risk of death due to prematurity-associated conditions is therefore an indicator of how women in a given country have access to safe and effective, pre- and post-natal medical care, as well as an indicator of the overall health of their society.

Large pooled analyses using data from multiple countries showed that the incidence of preterm birth has increased in recent years, with preterm birth an important risk factor for neonatal mortality [8, 9]. However, these analyses did not include data from China. One study analyzing the new-born information covering 2,377 monitoring sites in China showed that preterm birth complications are an important cause of child mortality in China [10]. The estimated number of preterm births (<37 weeks) was more than 250,000 in 2010 in China, ranking the second country behind India with the highest number of annual preterm births [11].

Economic development and government programs in China over the past two decades have together led to improved quantity and quality of maternal, child and newborn health care [11]. Secular trends in improved neonatal outcomes have not been well studied, however, including whether improved medical care has been evident in rural as well as rural settings [12]. Hubei Province is a large, economically, agriculturally and ethnically diverse province located in south-central China with approximately 57 million inhabitants. The aim of this study was therefore to describe secular trends in the incidence of premature birth as well as infant mortality linked with preterm birth in Hubei Province from 2001 to 2012, and to identify risk factors associated with these important health outcomes.

## Methods

### Study design

A random sampling stratified by urban and rural areas was conducted in Hubei Province, China. Sixteen counties were selected randomly by zip code from 9 urban areas and 13 rural areas as the monitoring sites, including 6 urban areas and 10 rural areas.

### Data collection

The annual number of live infant births at each of the monitoring sites was recorded by trained staff of the Maternal and Child Health Care Network in Hubei Province from January 1, 2001 to December 31, 2012.The data were collected based on an electrical “three vertical level” health care system in Hubei Province. The “three vertical levels” include community/countryside, city, and province.

Upon the birth of each infant within the monitoring sites, the basic information of the mothers was collected in face-to-face interview before hospital discharge by a licensed and trained health care provider in each county. The basic information was recorded electronically through the health care system, which included maternal age, education, income, occupation, pregnancy parity, birth parity, residence, use of Newborn Emergency Transport Service, infant gender, and use of assisted reproductive technology. The gestational week was assessed by trained doctors based on early pregnancy symptoms, pregnancy tests, and B-mode ultrasound tests. The birth weight was measured by trained doctors after the infant was delivered. Electronic weighing scales were used to weigh the infants, and the accuracy of weight was to 0.01 kg.

After the basic information was collected, health care providers in the city to which the counties belong reviewed and verified the total number of live births, preterm births, and preterm mortality. If the preterm infant was lost to follow-up within the first year after the infant’s birth due to migration, the migrated infant was identified by the connected health care system of the monitoring sites in other provinces of China.

Finally, the data on annual births within each city was reported to the Hubei Provincial Maternity and Child Care Hospital (HBPMCCH). Validation studies on the underreport rate were conducted by randomly selecting one of the monitoring sites.

HBPMCCH doctors independently collected and verified the data collected from the county health providers, and compared the data to reported data in the health care system. The underreported rate of live births decreased from 4.5 % (4.67 % in rural areas and 0.23 % in urban areas) in 2001 to 0.54 % (0.69 % in rural areas and 0.11 % in urban areas) in 2012. Written informed consent was obtained from at least one parent, and the study was approved by the Ethics Committee of Hubei Maternal and Child Health Hospital.

### Definition of preterm birth incidence and mortality of preterm birth

Live birth refers to the complete expulsion or extraction from its mother of a product of conception, irrespective of the duration of the pregnancy [13]. Preterm birth is defined as all births less than 37 whole weeks of gestation or fewer than 259 days since the first day of a woman’s last menstrual period, according to the WHO standards [3]. In accordance with the International Statistical Classification of Diseases and Related Health Problems, 10th Revision (ICD-10), preterm death was defined as death in the first year of life resulting from any cause related to preterm birth, but not from accidental causes [14]. Incidence of preterm birth was calculated as the ratio of the number of preterm live births to the number of total live births. Incidence of preterm mortality was calculated as the ratio of the number of preterm deaths to the number of preterm live infants.

### Statistical Analysis

To compare the difference of proportions between groups, a Chi-Square test was used with *P* < 0.05 indicating significant difference. To identify factors significantly associated with incidence of preterm birth and mortality, we initially included all covariates into the model and used logistic regression with backward stepwise selection. Candidate variables in the logistic model included year (continuous), maternal age (<35 y vs. ≥ 35 y), education (>9 years vs. ≤ 9 years), occupation (physical labor vs. office job), birth weight (<2.5 kg vs. ≥ 2.5 kg), pregnancy parity (1 vs. ≥ 2), birth parity (1 vs. ≥ 2), assisted reproductive technology (yes vs. no), income (<500 USD/month vs. ≥ 500 USD/month), residence (rural vs. urban), Newborn Emergency Transport Service (yes vs. no), infant gender (female vs. male), and gestational week (<34 week vs. ≥ 34 week). The maternal age, gestational week, and birth weight were dichotomized based on the literature. Education, pregnancy parity, birth parity, and income were dichotomized, with the largest number of participants as the reference group. For the variables with missing values, a missing indicator was used. The data was analyzed using the Statistical Package for the Social Sciences (SPSS) version 11.5.

## Results

### The overall preterm incidence and mortality rates of preterm birth

**Fig. 1:**
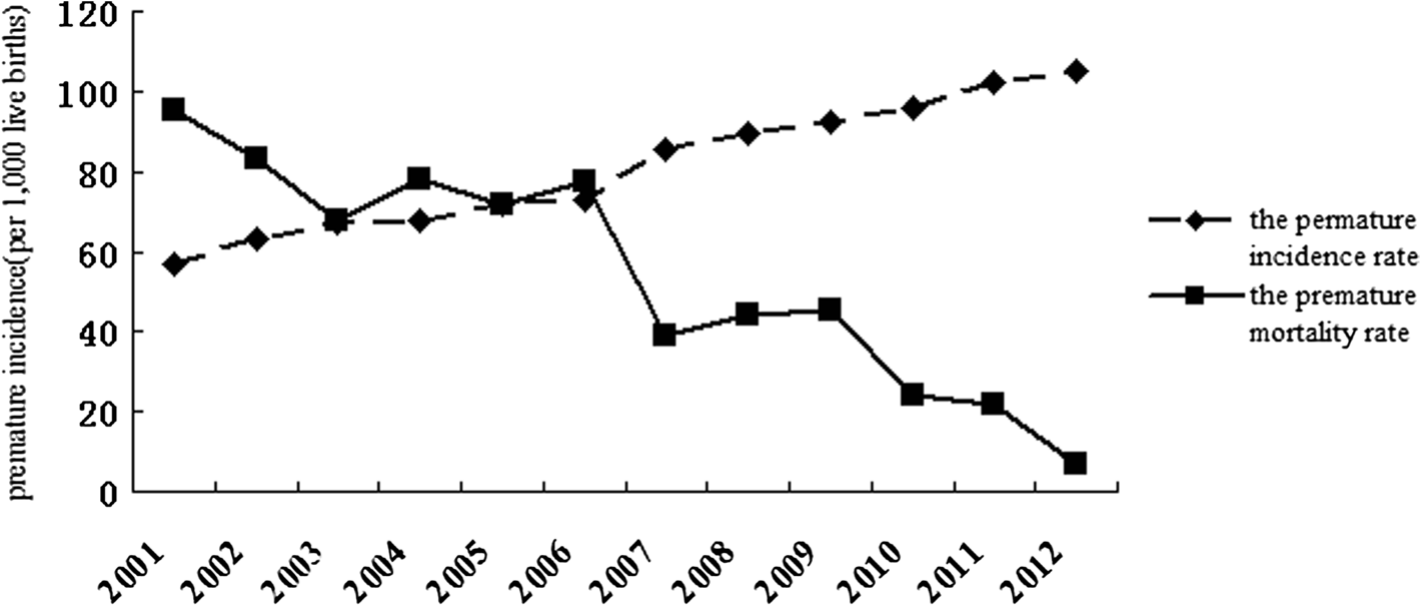
Annual incidence and mortality of preterm birth in Hubei Province from 2001 to 2012. P value was obtained by fitting logistic models with year (continuous) as the independent variable

A total of 818,481 live births were documented in the 16 survey areas in Hubei Province between 2001 and 2012. The incidence of preterm birth increased from 56.7 per 1,000 live births in 2001 to 105.2 per 1,000 live births in 2012, while the incidence of preterm mortality decreased from 95.1 per 1,000 live births in 2001 to 13.4 per 1,000 live births in 2012 (Fig. 1).

### The incidence of preterm birth in urban and rural areas

**Fig. 2:**
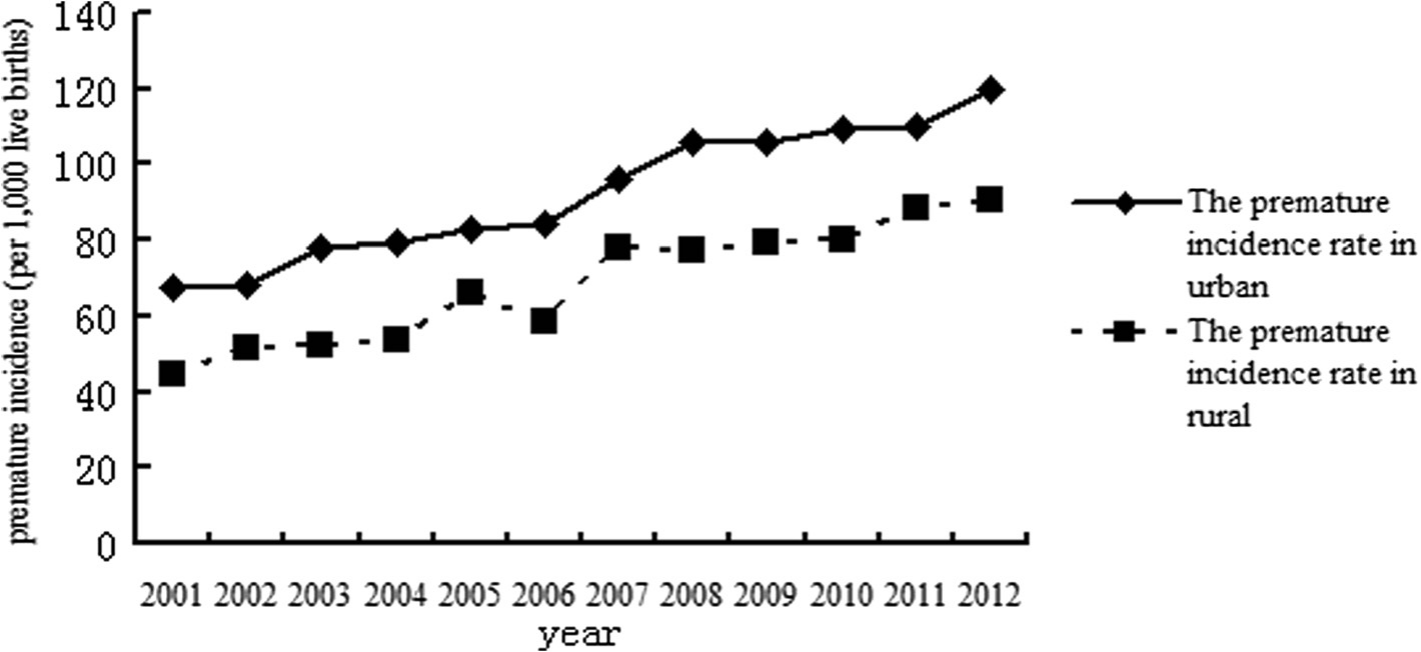
The incidence rates of preterm birth in urban and rural areas in Hubei Province from 2001 to 2012

Of the 76,923 preterm infants, 45,292 were in urban areas, and 31,631 were in rural areas. Preterm incidence increased steadily from 2001 to 2012 in both urban and rural areas. Preterm incidence rate in 2012 was almost 2 times the preterm incidence rate in 2001 in both urban and rural areas (Fig. 2). The incidence of preterm birth in the rural areas was lower than in the urban areas for each year. However, the trends between the rural and urban areas appeared to be similar.

**Fig. 3:**
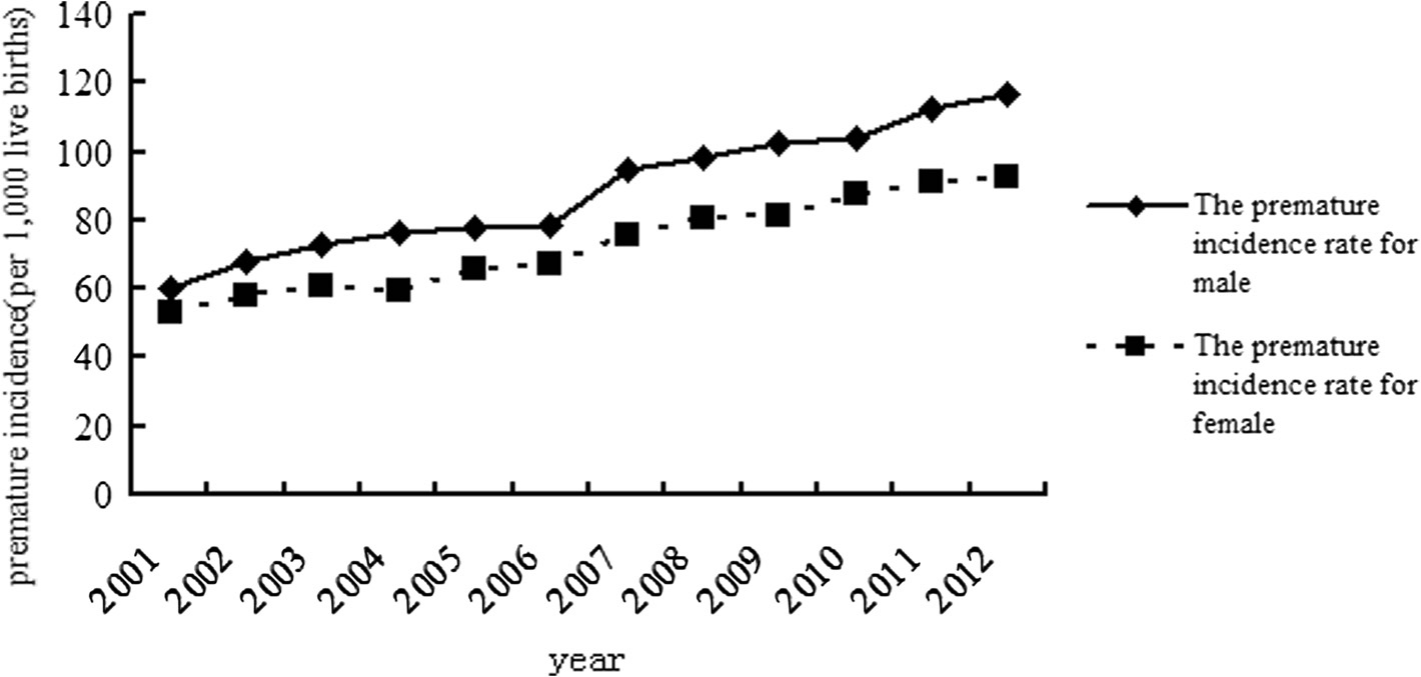
The incidence of preterm birth for males and females in Hubei Province from 2001 to 2012

The incidence of preterm birth for both genders increased steadily from 2001 to 2012. The trends for males and females appeared to be similar (Fig. 3).

### The mortality of preterm birth in urban and rural areas

**Fig. 4:**
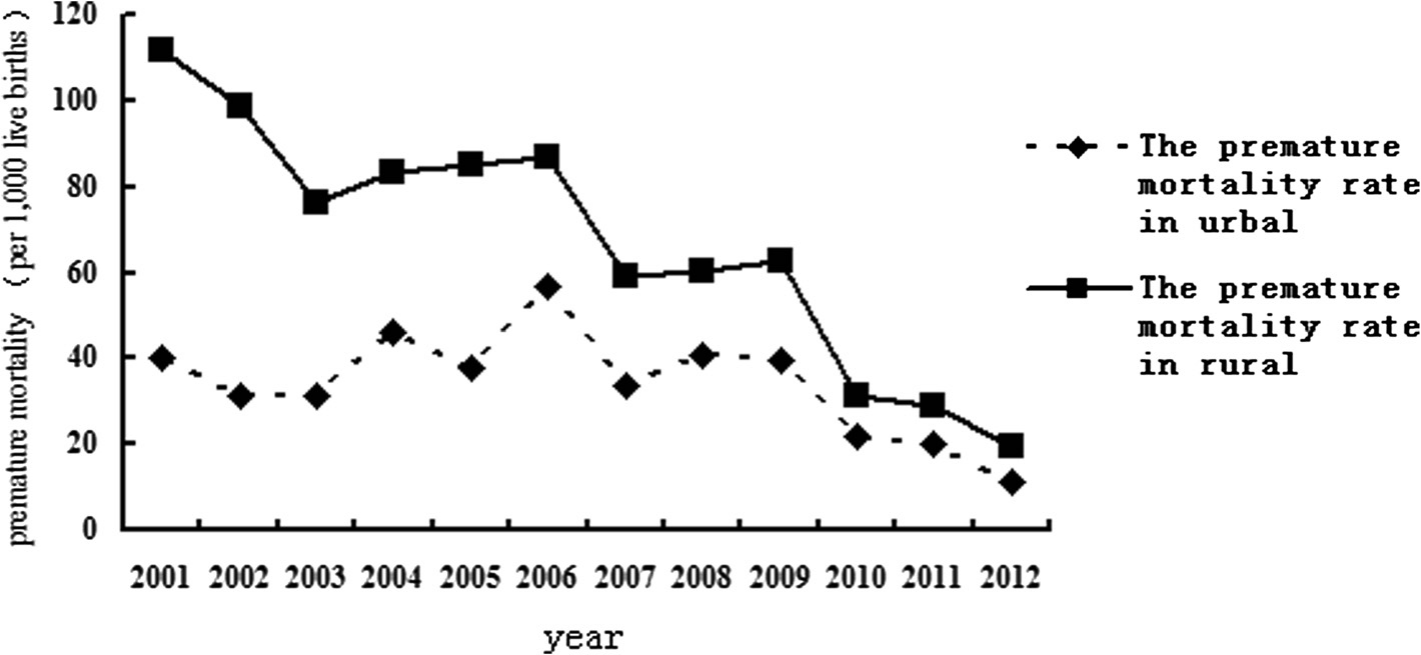
Changes in infant mortality rate due to prematurity in urban and rural areas in Hubei Province from 2001 to 2012

Of the 2,248 preterm deaths, 1,025 were in rural areas, and 1,123 were in urban areas. The mortality of preterm birth in rural areas decreased from 2001 to 2012, although an inverse V-shaped fluctuation pattern was observed. The mortality of preterm birth in 2012 was almost one-tenth the incidence in 2001. The mortality of preterm birth in urban areas also showed a downward trend with fluctuation from 2001 to 2012. The downward trend of mortality of preterm birth was steeper for rural areas than for urban areas, with the convergence of the two curves around 2012 (Fig. 4).

**Fig. 5:**
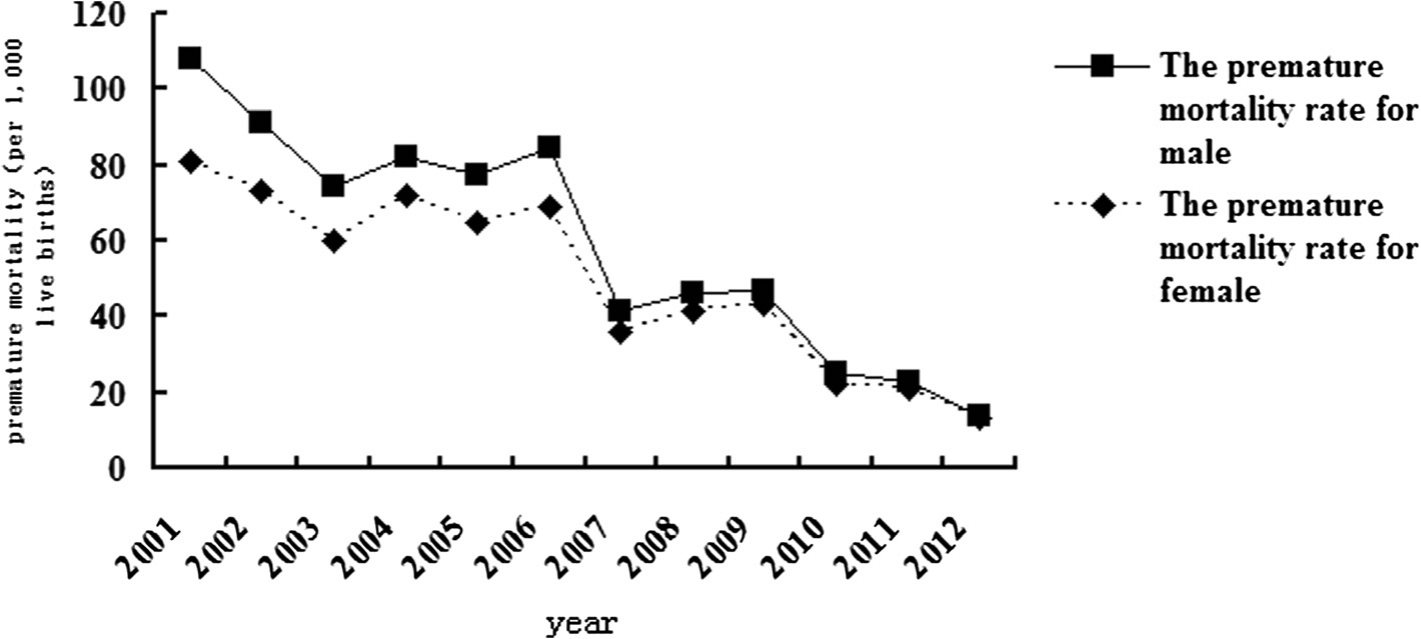
Changes in infant mortality rate due to prematurity for males and females in Hubei Province from 2001 to 2012

The overall trend and fluctuation patterns for both males and females were similar to Fig. 4. The preterm mortality rate was higher for males than females from 2001 to 2006 and converged thereafter (Fig. 5).

### Factors significantly associated with the incidence and mortality of preterm births

Characteristics of the covariates of the study population were shown in Table 1. We firstly performed univariate analysis to assess the associations between covariates shown in table 1 with risks of preterm birth and preterm mortality (Table 1, Table 2). After backward stepwise selection, older age of delivery, lower level of education, ART use, higher income, residence in urban areas, and being male were associated with significantly higher incidence of preterm birth (Table 2). Insufficient gestational week, lower birth weight, and lower income were associated with significantly higher mortality of preterm birth, while improved NETS were associated with significantly lower mortality of preterm birth (Table 3).

**Table 1:** Characteristics of the covariates according to outcome status of preterm birth and preterm mortality

	Number of live birth	Number of preterm birth	Number of deaths due to prematurity	Preterm birth rate (%)	Preterm mortality rate (‰)
Number of participants	818481	76923	2248	9.40	2.75
Maternal age (years)					
<35	580486	27693	961	4.77	1.66
35+	237995	49230	1287	20.69	5.41
Gestational week (weeks)					
<34	189486	23077	1753	12.18	9.25
34+	628995	53846	495	8.56	0.79
Birth weight (kg)					
<2.5	344491	43994	1674	12.77	4.86
2.5+	473990	32929	574	6.95	1.21
Pregnancy parity					
1	94901	43808	1471	46.16	15.50
1+	723580	33115	777	4.58	1.07
Birth parity					
1	61375	31638	987	51.55	16.08
1+	757106	45285	1261	5.98	1.67
Assisted reproductive technology (Yes)					
Yes	30959	23469	756	75.81	24.40
No	787522	53454	1492	6.79	1.90
Income (USD/month)					
<500	418798	50392	1577	12.03	3.76
500+	399683	26531	671	6.64	1.68
Education					
College	74060	3315	68	4.48	0.91
Other	744421	73608	2180	9.89	2.93
Residence					
Urban	350154	45300	1123	12.94	3.21
Rural	468327	31623	1125	6.75	2.40
Newborn Emergency Transport Service					
Yes	12755	5562	48	43.60	3.75
No	805726	71361	2200	8.86	2.73
Infant gender					
Male	415496	44346	1348	10.67	3.24
Female	402985	32577	900	8.08	2.23
Occupation					
Office job	447921	20562	477	4.59	1.07
Other	370560	56361	1771	15.21	4.78
Single birth					
Yes	787168	73715	2229	9.36	2.83
No	31313	3208	19	10.24	0.60

**Table 2:** Regression analysis identifying factors associated with preterm birth in Hubei Province, China (2001 – 2012)

Covariates	Univariate analysis OR (95 % CI)	Multivariable analysis* OR (95 % CI)
Year	1.01 (1.00 - 1.01)	----
Maternal age (<35 y vs. ≥ 35 y)	5.28 (5.04 - 5.45)	0.57(0.55-0.58)
Gestational week(<34 week vs. ≥ 34 week)	2.21 (1.86 - 2.63)	----
Birth weight(<2.5 kg vs. ≥ 2.5 kg)	1.08 (1.05 -1.13)	----
Pregnancy parity(1 vs. ≥ 2)	2.80 (2.69 - 2.95)	----
Birth parity (1 vs. ≥ 2)	1.08 (1.01 - 1.10)	----
Assisted reproductive technology (yes vs. no)	1.97 (1.91 -2.05)	3.67 (3.56-3.78)
Income (<500 USD/month vs. ≥ 500 USD/month)	1.27 (1.22- 1.32)	0.63 (0.61-0.66)
Education (>9 years vs. ≤ 9 years)	0.71 (0.49 - 0.91)	1.04 (1.01-1.06)
Residence (rural vs. urban)	1.37 (1.35 - 1.39)	1.27 (1.24-1.30)
Newborn Emergency Transport Service (yes vs. no)	3.13 (3.02 - 3.23)	----
Infant gender (female vs. male)	0.59 ( 0.56 - 0.63)	3.31 (3.23 -3.39)
Occupation (physical labor vs. office job)	2.16 (2.09 - 2.23)	----
Single birth	0.91 (0.89 - 0.97)	----

*Multivariate logistic model was fit with all characteristics considered as predictors of preterm birth, and a backward-selection procedure was used to select significant variables included in the final model, with a *P* value < 0.05 indicating significance

**Table 3:** Regression analysis identifying factors associated with preterm mortality in Hubei Province, China (2001 – 2012)

Covariates	Univariate analysis OR (95 % CI)	Multivariable analysis* OR (95 % CI)
Year	1.00 (1.00 -1.01)	----
Maternal age (<35 y vs. ≥ 35 y)	1. 32 (1. 22–1.51)	----
Gestational week (<34 week vs. ≥ 34 week)	2.76 ( 1.92 -2.99)	1.01 (1.00-1.02)
Birth weight (<2.5 kg vs. ≥ 2.5 kg)	1.07 ( 1.02 - 1.11)	1.02 (1.00-1.03)
Pregnancy parity (1 vs. ≥ 2)	1.08 (1.08 -1.11)	----
Birth parity (1 vs. ≥ 2)	1.00 (1.00 - 1.01)	----
Assisted reproductive technology (yes vs. no)	1.13 (1.09-1.15)	----
Income (<500 USD/month vs. ≥ 500 USD/month)	1.31 (1.27 -1.39)	1.12 (1.01-1.22)
Education (>9 years vs. ≤ 9 years)	0.71 (0. 49–0. 89)	----
Residence (rural vs. urban)	1.14 ( 1.10 - 1.29)	----
Newborn Emergency Transport Service (yes vs. no)	2. 14 (2.00 - 3.02)	0.81 (0.77-0.99)
Infant gender (female vs. male)	0.67 ( 0.51 - 0.74)	----
Occupation (physical labor vs. office job)	1.18 (1.10 -1.23)	----
Single birth	0.97 (0.95 - 0.99)	----

*Multivariate logistic model was fit with all characteristics considered as predictors of preterm mortality, and a backward-selection procedure was used to select significant variables included in the final model, with a *P* value < 0.05 indicating significance

### Sensitivity analysis

Given that plurality birth might be an important risk factor of preterm birth and preterm mortality [15], we conducted stratified analysis by singletons and multiples on the risk factors of preterm birth and preterm mortality. The risk factors identified by backward stepwise selection did not change within each stratum (Additional file 1: Table S1–S4).

## Discussion

The incidence rate of preterm birth increased gradually from 56.7 to 105.2 per 1,000 live births, which was comparable to the overall preterm birth rate in China (7.1 %) in 2011 [16], while the incidence of preterm mortality decreased remarkably from 95.1 to 13.4 per 1,000 live births from 2001 to 2012 in Hubei Province, China. In this large sample, we identified significant predictors of incidence and mortality of preterm births.

Preterm birth occurs for a variety of reasons. Early induction of labor or cesarean birth, either for medical or non-medical reasons are causes of some of the preterm births. Most preterm births occur spontaneously [17]. Multiple pregnancies, infections, and chronic conditions such as diabetes and high blood pressure are common established causes. In addition, the use of ovulation inducing medications and intrauterine infections may also have contributed to the rise in the preterm birth rate.

Our study showed that the incidence and mortality of preterm birth differed in urban and rural areas. The regions in Hubei Province were divided into urban and rural areas according to the local economic development and population density of these areas. From 2001 to 2012, the incidence of preterm birth in urban areas was about 1.5 times that of rural areas, while the incidence of preterm mortality decreased faster in urban areas than in rural areas. The results of this study showed that premature incidence and mortality were associated with social-economic development of the area. Our results were similar to a study in North Carolina which found incidence of preterm birth was more pronounced in urban residents [18]. Economic development is not well balanced in Hubei Province. Our study found that higher income was a risk factor of preterm birth but a protective factor for the mortality of preterm birth. The shift to an urban lifestyle leads to increased work pressure, a delay of child-delivering age, and increased adolescent pregnancy (early marriage) risk, which are all factors associated with high rates of preterm births [19]. Previous studies have shown that preterm delivery often occurs in women aged <19 years old or >35 years old [20]. Our study found that the incidence of preterm birth was positively associated with maternal age (years) ≥35. In addition, preterm incidence was significantly higher in women who used assisted reproductive technology for conception than women with natural conception [21]. Seven hospitals in urban areas were allowed to carry out human assisted reproductive technology in Hubei. This may explain the disparity of the incidence of preterm birth between urban and rural areas in the province.

In our study, except for in 2012, preterm mortality in urban areas was lower than rural areas. Although a great achievement has been made in reducing preterm mortality in Hubei Province, it is still relatively high in poor areas. Our study found that preterm mortality risk is lower for residents in urban areas. In fact, most preterm deaths and disabilities attributable to childbirth are avoidable, as there are medical solutions to prevent or manage preterm-related complications that cause preterm death. Thus, the establishment and improvement of the three-level health care network for women and children in China has proven to be effective and essential for immediate access to high-quality maternal health care both in rural and urban areas [22, 23].

From 2001 to 2012, premature incidence and mortality for males were higher than females, consistent with previous studies. Several mechanisms have been proposed to explain why pregnancies carrying male fetuses could have a higher risk of preterm birth. First, heavier body weight of the male fetus increases the probability of using preterm labor [24]. Second, there is a greater susceptibility to gestational hypertension or infection which are associated with preterm birth. Third, male and female fetuses may have different sex-linked biochemical processes, including estrogen production from androgen precursors or by interleukin-1 [25]. In addition, male infants are more likely to have lower Apgar scores, and more likely to have respiratory distress syndrome or lung related injuries and disabilities [24].

Our study identified several factors that were associated with incidence and mortality of preterm birth. Unhealthy habits and lack of health knowledge are factors associated with preterm infant deaths [26]. It is essential that elementary obstetric and neonatology health providers are professionally trained in order to increase their capacity to successfully manage severe newborn complications such as intracranial hemorrhage [27]. Higher total expenditures on health per capita are one of the factors associated with lower preterm mortality. Other important factors associated with low hospitalized delivery rates include inability to pay for expenditure of health care, and inconvenient transportation in rural and mountain areas, which delays the transfer of severely sick neonates among hospitals.

Our study found that higher preterm mortality risk is associated with lower per capita GDP. In poor areas, most of the preterm infants died at home immediately after leaving the delivery hospital. Some preterm infants born in low-income regions die within their first few days of life. In poor regions, even for those born in a clinic or hospital, primary neonatal care is often lacking [28]. The risk of a neonatal death due to complications of preterm birth is higher in poor than rich regions [29]. To reduce preterm birth, a priority system should be set up for tracking and providing emergency treatment to preterm children.

With rapid socio-economic development, as well as improvements in obstetrics and neonatal rescue technology, the neonatal transport network was set up to decrease preterm child mortality. Our study found that lower preterm mortality risk was associated with improved newborn emergency transport services. Essential newborn car includes thermal care, breastfeeding support, and infection prevention and management and, if needed, neonatal resuscitation. Extra care for small babies, such as Kangaroo Mother Care program (e.g., carrying the baby skin-to-skin, and additional support for breastfeeding), was estimated to save approximately 450,000 babies each year [30]. Care for preterm babies with complications includes treating infections with antibiotics, safe oxygen management, supportive care for respiratory distress syndrome, continuous positive airway pressure and/or surfactant, and neonatal intensive care for those regions with lower mortality and higher health system capacity [31].

There were several potential limitations in our study. First, we did not differentiate iatrogenic and spontaneous preterm birth. The higher rate of preterm birth over time might be due to the advanced technologies, such as induction of labour and cesarean section. It has been shown that in the United States, the increase of cesarean birth is in part responsible for the overall increase in the preterm birth rate from 1990 to 2007 and the decline in perinatal mortality [32]. Second, the ultrasound dating (USS) is more widely used to estimate gestational age nowadays instead of latest menopausal period (LMP), which has low accuracy due to the considerable variation in the length of menstrual cycle among women. A study conducted in Canada showed that the USS was associated with a higher rate of preterm birth than LMP [33]. However, we did not obtain detailed data on the estimation method of prematurity. Third, our preterm birth and infant mortality only included live births, and stillbirth was not taken into account [34]. Thus, the incidence and mortality rate of preterm birth might be underestimated in our study. Fourth, stillbirth in less developed areas might be turned into live birth in more developed areas, and this might contribute to the higher incidence of live birth in urban than rural areas. However, this phenomenon, even if true, could not explain the increasing trend of preterm birth in both urban and rural areas over time.

## Conclusions

In summary, an increase in the incidence of preterm birth and a decrease in the mortality rate of preterm birth were observed in Hubei Province from 2001 to 2012. Our results showed that delivery at an advanced maternal age, use of ART, and the lack of NETS were associated with increased incidence and mortality of preterm birth. Our results provide important information for areas of improvements in reducing incidence and mortality of premature birth.

## Additional file

Additional file 1: Table S1. Multivariable regression analysis identifying factors associated with preterm birth in Hubei Province, China from 2001 to 2012 among single birth infants. **Table S2.** Multivariable regression analysis identifying factors associated with preterm mortality in Hubei Province, China (2001 – 2012) among single birth infants. **Table S3.** Multivariable regression analysis identifying factors associated with preterm birth in Hubei Province, China from 2001 to 2012 among multiple birth infants. **Table S4.** Multivariable regression analysis identifying factors associated with preterm mortality in Hubei Province, China (2001 – 2012) among multiple birth infants. (DOCX 20 kb)

## Abbreviations

ART: Assisted reproductive technology
GDP: Gross domestic production
NETS: Newborn emergency transport services
