# Supplementary material for: Time trends and risk factor associated with premature birth and infants deaths due to prematurity in Hubei Province, China from 2001 to 2012
Source: BMC Pregnancy Childbirth. 2015 Dec 10;15:329. doi: 10.1186/s12884-015-0767-x (PMC4676169; doi:10.1186/s12884-015-0767-x)
Supplement: Additional file 1: Table S1. — Multivariable regression analysis identifying factors associated with preterm birth in Hubei Province, China from 2001 to 2012 among single birth infants. Table S2. Multivariable regression analysis identifying factors associated with preterm mortality in Hubei Province, China (2001 – 2012) among single birth infants. Table S3. Multivariable regression analysis identifying factors associated with preterm birth in Hubei Province, China from 2001 to 2012 among multiple birth infants. Table S4. Multivariable regression analysis identifying factors associated with preterm mortality in Hubei Province, China (2001 – 2012) among multiple birth infants. (DOCX 20 kb) [file 12884_2015_767_MOESM1_ESM.docx]

Supplemental table 1. Multivariable regression analysis identifying factors associated with preterm birth in Hubei Province, China from 2001 to 2012 among single birth infants.

| Variable (reference group) | OR (95%Cl ) | P |
| --- | --- | --- |
| Maternal Age (< 35 y) | 0.65 (0.62 - 0.68) | <.0001 |
| Education (≤ 9 years) | 1.17 (1.13 -1.22) | <.0001 |
| Assisted Reproductive Technology (ART) (No) | 3.42 ( 3.26 - 3.58) | <.0001 |
| GDP (<500 USD/month) | 0.43 ( 0.37 - 0.49) | <.0001 |
| Residence (rural) | 2.24 ( 2.16 - 2.33) | <.0001 |
| Gender (male ) | 2.73 (2.63- 2.84) | <.0001 |

Multivariate logistic model was fit with all characteristics considered as predictors of preterm birth, and a backward-selection procedure was used to select significant variables included in the final model, with a P value < 0.05 indicating significance.

Supplemental table 2. Multivariable regression analysis identifying factors associated with preterm mortality in Hubei Province, China (2001 – 2012) among single birth infants.

| Variable (reference group) | OR (95%Cl ) | P |
| --- | --- | --- |
| Gestational week (＜34 week) | 1.01 (1.00 - 1.02) | <.0001 |
| Birth weight (＜2.5 kg) | 1.02 (1.00 - 1.03) | <.0001 |
| Newborn Emergency Transport Services (No) | 0.84 (0.70 - 0.99) | <.0001 |
| GDP (< 500 USD) | 1.61 (1.34 - 1.95) | <.0001 |

Multivariate logistic model was fit with all characteristics considered as predictors of preterm mortality, and a backward-selection procedure was used to select significant variables included in the final model, with a P value < 0.05 indicating significance.

Supplemental table 3. Multivariable regression analysis identifying factors associated with preterm birth in Hubei Province, China from 2001 to 2012 among multiple birth infants.

| Variable (reference group) | OR (95%Cl ) | P |
| --- | --- | --- |
| Maternal Age (< 35 y) | 0.54 ( 0.49 - 0.59) | <.0001 |
| Education (≤ 9 years) | 1.20 (1.00 - 1.45) | 0.046 |
| Assisted Reproductive Technology (ART) (No) | 3.55 (3.27 - 3.85) | <.0001 |
| GDP (<500 USD/month) | 0.52 ( 0.47 - 0.58) | <.0001 |
| Residence (rural) | 2.05 (1.93 - 2.18) | <.0001 |
| Gender (male ) | 2.75 (2.56 - 2.93) | <.0001 |

Multivariate logistic model was fit with all characteristics considered as predictors of preterm birth, and a backward-selection procedure was used to select significant variables included in the final model, with a P value < 0.05 indicating significance.

Supplemental table 4. Multivariable regression analysis identifying factors associated with preterm mortality in Hubei Province, China (2001 – 2012) among multiple birth infants.

| Variable (reference group) | OR (95% CI ) | P |
| --- | --- | --- |
| Gestational week (＜34 week) | 1.01 (1.00 -1.02) | <.0001 |
| Birth weight (＜2.5 kg) | 1.02 (1.01 - 1.04) | <.0001 |
| Newborn Emergency Transport Services (No) | 0.70 (0.60 - 0.90) | <.0001 |
| GDP (< 500 USD) | 1.29 (1.02 - 1.41) | <.0001 |

Multivariate logistic model was fit with all characteristics considered as predictors of preterm mortality, and a backward-selection procedure was used to select significant variables included in the final model, with a P value < 0.05 indicating significance.
